# Antibacterial activity of Cu(II) and Co(II) porphyrins: role of ligand modification

**DOI:** 10.1186/s13065-020-00701-6

**Published:** 2020-08-14

**Authors:** Belete B. Beyene, Getaneh A. Wassie

**Affiliations:** Department of Chemistry, Bahir Dar University, P. O. Box 79, Bahir Dar, Ethiopia

**Keywords:** Cobalt porphyrin, Inhibition, Copper porphyrin, Antibacterial activity, Lipophilicity

## Abstract

In this study, we report antibacterial activity of metalloporphyrins; 5, 10, 15, 20-tetrakis (*para*-X phenyl)porphyrinato M (II) [where X = H, NH_2_ and COOMe for M = Cu and X = COOH  and OMe for M = Co]. The activity study of the as-synthesized metalloporphyrins toward two Gram-positive (*S. aureus* and *S. pyogenes)* and two Gram-negative (*E. coli* and *K. pneumoniae*) bacteria showed a promising inhibitory activity. Among the complexes under study, the highest antibacterial activity is observed for 5, 10, 15, 20-tetrakis (*p*-carboxyphenyl)porphyrinato cobalt (II), with inhibition zone of 16.5 mm against Staphylococcus aureus (*S. aureus*). This activity could be attributed to the high binding ability of COOH group to cellular components, membranes, proteins, and DNA as well as the lipophilicity of the complex. Moreover, consistent with literature report, the study revealed that metalloporphyrins with electron withdrawing group at para-positions have better antibacterial activity than metalloporphyrin which possess electron donating group at para position.

## Introduction

Metalloporphyrins are assumed to have extra ordinary importance in recent years as agents for photodynamic therapy, optoelectronic devices, sensors, molecular logic devices and artificial solar energy harvesting and storage schemes [1]. Taking into account a great number of infections resulting from different bacterial species and the growing antibacterial resistance, the development of compounds with high antibacterial activities and novel mechanism of action is an urgent need [2–4]. As a consequence, researchers are designing novel, convenient, robust and inexpensive strategies for combating microorganisms with minimal invasive consequences [5, 6]. In this regard, natural and synthetic metalloporphyrins are among relatively low toxic molecules (either in vitro or in vivo) and are capable of effecting microbial and viral pathogens through the large number of different mechanisms [7]. In addition, the possibility of structural modifications place these molecules into a group of compounds that present a sustainable source for discovery of novel procedures, materials and agents active against a wide range of pathogenic microorganisms [7]. Modification of porphyrin ligand at the peripheral positions provokes tunable shape, size and symmetry which have suitable applications in materials and therapeutics [8]. The most common structural modification of synthetic porphyrins is made at the meso-position to achieve target molecules with required properties in biomedical applications such as photo diagnosis, cancer therapy and as antibacterial agents [9, 10]. Nowadays, an ever increase in the mortality rate throughout the world is linked with infectious diseases with multiple resistances to antibiotics and the lack of effective treatments [2–4].

Porphyrin based systems have been reported as potential antibacterial agents against Gram-positive and Gram-negative bacteria species for decades [11–21]. They were used to treat different kinds of bacteria including bacillus subtilis, *Escherichia coli*, mycobacterium smegmatis, and actinobacillus [22–24]. The activities are based on their ability to catalyse peroxidase–oxidase reactions, generate reactive oxygen species (ROS) by absorbing light and partition into lipids of bacterial membranes [2, 25]. However, in most cases, much attention has been paid to ionic porphyrins (cationic [4, 15, 16, 26–33] and anionic [34–36] presumably because of their ability to strongly bind with cellular components and better activity than the neutral ones [15, 28, 37]. But, ionic porphyrins are very limited and studies involving neutral porphyrins to treat bacterial infections are becoming attractive. In line with this, antibacterial activity has been reported against *Staphylococcus aureus*, Mycobacterium smegmatis and Yersinia enterocolitica by using neutral porphyrins with the alkyl substituents at the β-pyrrolic positions [15, 33, 38]. However, there is no intensive report or documantation regarding neutral porphyrins for treating antibacterial infections.

**Fig. 1:**
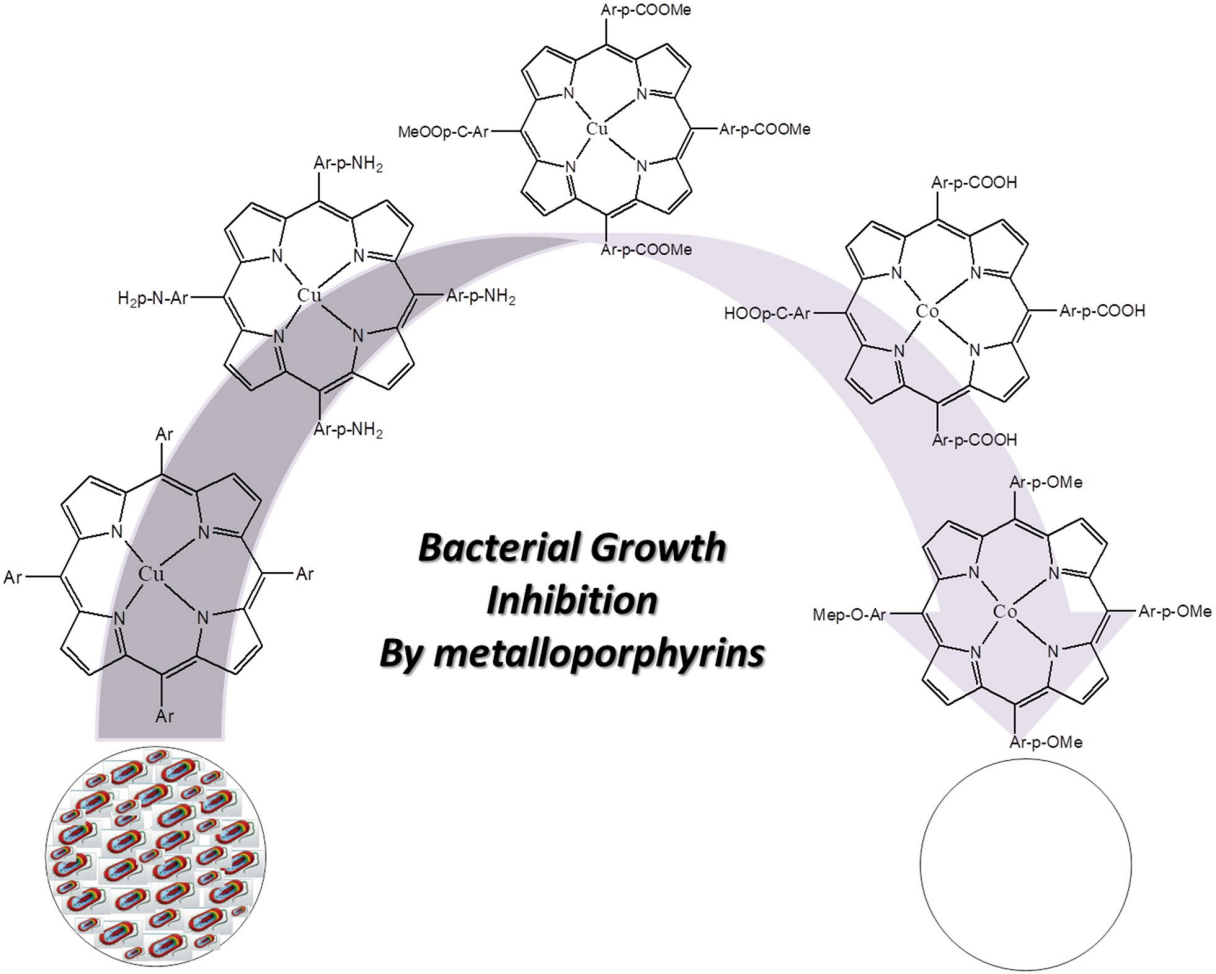
A schematic diagram for bacterial growth inhibition by metalloporphyrins

In this work we, therefore report antibacterial activity of 5, 10, 15, 20-tetrakis (*para*-X phenyl)porphyrinato M (II) [where X = H, NH_2_ and COOMe for M = Cu and X = COOH and OMe for M = Co] for the first time (Fig. 1). To the best of our knowledge the antibacterial activity of these particular metalloporphyrins is not reported previously. The study revealed the highest antibacterial activity for Co (II) porphyrin containing COOH, which could be attributed to the high binding ability of COOH group to various cellular components and its lipohilicity.

## Experimental

### Antibacterial activity testing

The metal salts, ligands and their metal complexes were evaluated for in vitro antibacterial activities against strains of the two Gram-negative bacterial strains such as *Escherichia coli* (*E. coli*) and *Klebsiella pneumoniae* (*K. pneumoniae*); two Gram positive bacterial strains such as *Staphylococcus aureus* (*S. aureus*) and *Streptococcus pyogenes* (*S. pyogenes*) bacterium by disc diffusion method. In this method, activity of the test compounds was expressed by measuring the diameter of zone of inhibition. The plates were observed for zones of inhibition after 24 h, and incubation at 37 °C. The diameters of the zone of inhibition produced by the complexes were compared with a standard antibiotic drug Gentamycin. All the bacterial strains used in the experiment were received from microbiology laboratory, Bahir Dar University.

### Media preparation and sterilization

The Culture media (Mueller Hinton) were prepared according to the manufacturer’s guideline (suspend 38 g in 1 L of distilled water). The mass of the culture medium was weighed and dissolved in distilled water. The mixture was stirred with a sterilized glass rod and tightly covered with an aluminum foil and then the culture medium was autoclaved for 15 min at 121 °C. Next to that,the agar was allowed to cool in order to maintain the media in a molten stage. Petri dishes were dried in lower humidity by keeping them in a laminar flow hood. The freshly prepared and cooled Muller–Hinton agar was spread at the surface of petri dishes.

### Inoculation of test plates

A small volume, about 0.1 mL of the bacterial suspensions were inoculated onto the dried surface of Muller–Hinton agar plate and streaked (swabbed) by the sterile cotton swab over the entire sterile agar surface. This procedure was repeated by streaking two more times, rotating the plate approximately 60 °C each times to ensure an even distribution of inoculums and the rim of the agar was swabbed. The lid was left ajar for 3–15 min, to allow for any excess surface moisture to be absorbed before applying the samples on the respective well.

### Sample injection and incubation

Anti-bactericidal activities of each reagents and synthesized complexes were evaluated by the disc diffusion method. Agar were prepared by using a sterilized cork borer with 6 mm diameter, 4 mm deep and about 2.5 cm apart to minimize overlapping of zones. Then holes of 6 mm diameter were punched carefully using a sterile cork borer. The metal salts of each complex, DMSO, the ligands, and their metal complexes were carefully injected to the respective disc in duplicate. The reference antibiotic agent disc (gentamycin) was dispensed via sterile pair of forceps onto the surface of the inoculated agar plate and pressed down to ensure complete contact with the agar surface. It was allowed to diffuse for about 40 min before incubation and then the plates were incubated at 37 °C for 24 h. After 24 h incubation, the antibacterial activity was evaluated by measuring the diameter of inhibition zones in millimeter. The test was carried out in duplicate and the results were recorded as mean ± standard deviation.

## Results and discussion

### Synthesis and photophysical properties

The metalloporphyrins employed in this study were synthesized by following reported methods [39–44]. The detail synthetic procedure, characterization data and photophysical properties of as synthesized compounds is shown in supporting information.

### Antibacterial activity

The as synthesized metalloporphyrins were tested for their in vitro antibacterial activity in the open condition under visible/white light and the results were compared with the ligand, metal salt and the commercially available drug, *gentamycin,*. The activity is 1st tested against two Gram-positive (*Staphylococcus aureus* (*S. aureus*) and *Streptococcus pyogenes *(*S. pyogenes*) and two Gram-negative [*Escherichia coli* (*E. coli*) and *Klebsiella pneumoniae* (*K. pneumoniae*)] bacteria by using 31.25, 62.5, 125, 250 and 500 mg/L of each metalloporphyrins. All the tested metalloporphyrins were found to be active against all the tested pathogens and compared with the commercially available antibiotic drug (gentamycin). The result of antibacterial activities is reported as inhibition zone diameter (mm) for the concentration of 500 mg/L as shown in Table 1.

**Table 1:** Antibacterial activiy (mean IZ diameter (mm) ± SD) of metalloporphyrins, corresponding ligands, metal salts, and gentamycin with concentration 500 mg/L

Compounds	Antibacterial activiy (mean IZ diameter (mm) ± SD)			
	*S. aureus*	*S. pyogenes*	*E. coli*	*K. pneumonia*
CoTPPCOOH	*16.5 ± 0.5*	15 ± 0.3	16 ± 0.5	16 ± 0.6
CoTPPOMe	12 ± 0.2	*14 ± 0.6*	10 ± 0.3	12 ± 02
CuTPPCOOMe	*16 ± 0.2*	15 ± 0.5	13 ± 0.6	13.5 ± 075
CuTPPNH_2_	13 ± 0.4	*13.5 ± 0.3*	12 ± 0.45	12.5 ± 0.6
H_2_TPPCOOH	8.5 ± 0.2	9 ± 0.3	7.5 ± 0. 3	7.5 ± 0.4
H_2_TPPCOOMe	8.25 ± 0.3	8 ± 0.2	7.5 ± 0.03	7.5 ± 0.2
H_2_TPPOMe	7 ± 0.3	7.25 ± 0.3	7 ± 0.3	7 ± 0.2
H_2_TPPNH_2_	7.5 ± 0.2	7.75 ± 0.3	7 ± 0.01	7 ± 0.2
CuCl_2_·2H_2_O	6.75 ± 0.2	7 ± 0.3	6.25 ± 0.02	6.5 ± 0.4
Co acetate	6.5 ± 0.1	7 ± 0.2	6.5 ± 0.1	6.5 ± 0.2
DMSO	0 ± 0.00	0 ± 0.00	0 ± 0.00	0 ± 0.00
10 µg gentamicin	25 ± 0.6	27 ± 0.75	26 ± 0.75	25 ± 0.5

All the complexes under study showed better antibacterial growth inhibition activity than the corresponding porphyrin ligand. This is a clear indication for the involvement of metal ions as potential candidates in bacterial growth inhibition. The justification for enormous antibacterial activity of transition metal complexes of porphyrins is based on overtone concept and chelation theory. The solubility of the complexes in lipid is an important factor to control the antibacterial activity [45–48]. Based on the overtone concept of cell permeability, the passage of the materials which are only lipophilic is favored by the lipid membrane that surrounds the cell. On the other hand, the dramatic decrease in polarity of metal ions because of an overlap of orbital of ligand and partial sharing of the positive charge of the metal ion with donor groups can be blearily explained by employing chelation theory. Moreover, this phenomenon increases the π-electron delocalization all over the whole porprhyrin ring and enhances the lipid solubility behavior of the complexes. Presumably, an increase in lipid-solubility of the porphyrin ligands upon metallation makes the complexes easily move across the bacterial cell. This process inhibits the metals to bind with the enzymes in microorganisms. In addition, the respiration process of the cell could be interrupted and thereby block the synthesis of biomolecules, which limit over enlargement of organism [49].

**Fig. 2:**
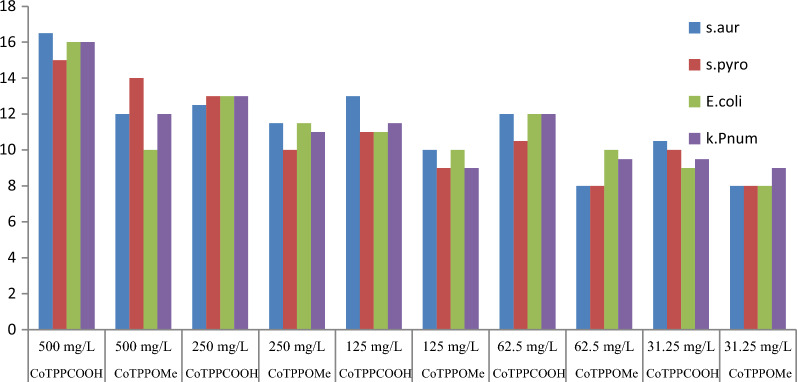
Bar graph of 5, 10, 15, 20-tetrakis (p-X phenyl)porphyrinato cobalt (II), where X = COOH and OMe on the same concentration pattern

As can be seen from Table 2 and Fig. 2 increasing the concentration of antibacterial agents increase the activity very slightly and metalooporphyrins under study are active and inhibit bacteria even at the lowest concentration (31.25 mg/L). Moreover, the bacteria growth inhibition activity of the complexes is not significantly different among different bacteria species. The 5, 10, 15, 20-tetrakis (*p*-carboxyphenyl)porphyrinato cobalt (II), exhibited the greater antimicrobial activities than other metalloporphyrins with inhibition zones 16.5 mm for *S. aureus* presumably attributing to its ability to strongly bind with cellular components.

**Table 2:** Antibacterial activiy (mean IZ diameter (mm) ± SD) of cobaltporphyrins, at different concentrations

Cobalt complexes at different concentration		Antibacterial activiy (mean IZ diameter (mm) ± SD)			
		*S. aureus*	*S. pyogenes*	*E. coli*	*K. pneumonia*
500 mg/L					
CoTPPCOOH		16.5 ± 0.5	15 ± 0.3	16 ± 0.5	16 ± 0.6
CoTPPOMe		12 ± 0.2	14 ± 0.6	10 ± 0.3	12 ± 02
250 mg/L					
CoTPPCOOH		12.5 ± 0.45	13 ± 0.3	13 ± 0.4	13 ± 0.3
CoTPPOMe		11.5 ± 0.3	10 ± 0.2	11.5 ± 0.2	11 ± 0.0.45
125 mg/L					
CoTPPCOOH		13 ± 0.3	11 ± 0.55	11 ± 0.3	11 ± 0.75
CoTPPOMe		10.5 ± 0.5	9 ± 0.5	10 ± 0.5	9 ± 0.3
62.5 mg/L					
CoTPPCOOH		12 ± 0.6	10.5 ± 0.4	12 ± 0.5	12 ± 0.4
CoTPPOMe		8 ± 0.2	8 ± 0.3	9 ± 0.4	9.5 ± 0.2
31.25 mg/L					
CoTPPCOOH		10.5 ± 0.3	10 ± 0.2	9 ± 0.1	9.5 ± 0.3
CoTPPOMe		8 ± 0.4	8 ± 0.2	8 ± 0.45	9 ± 0.3

**Fig. 3:**
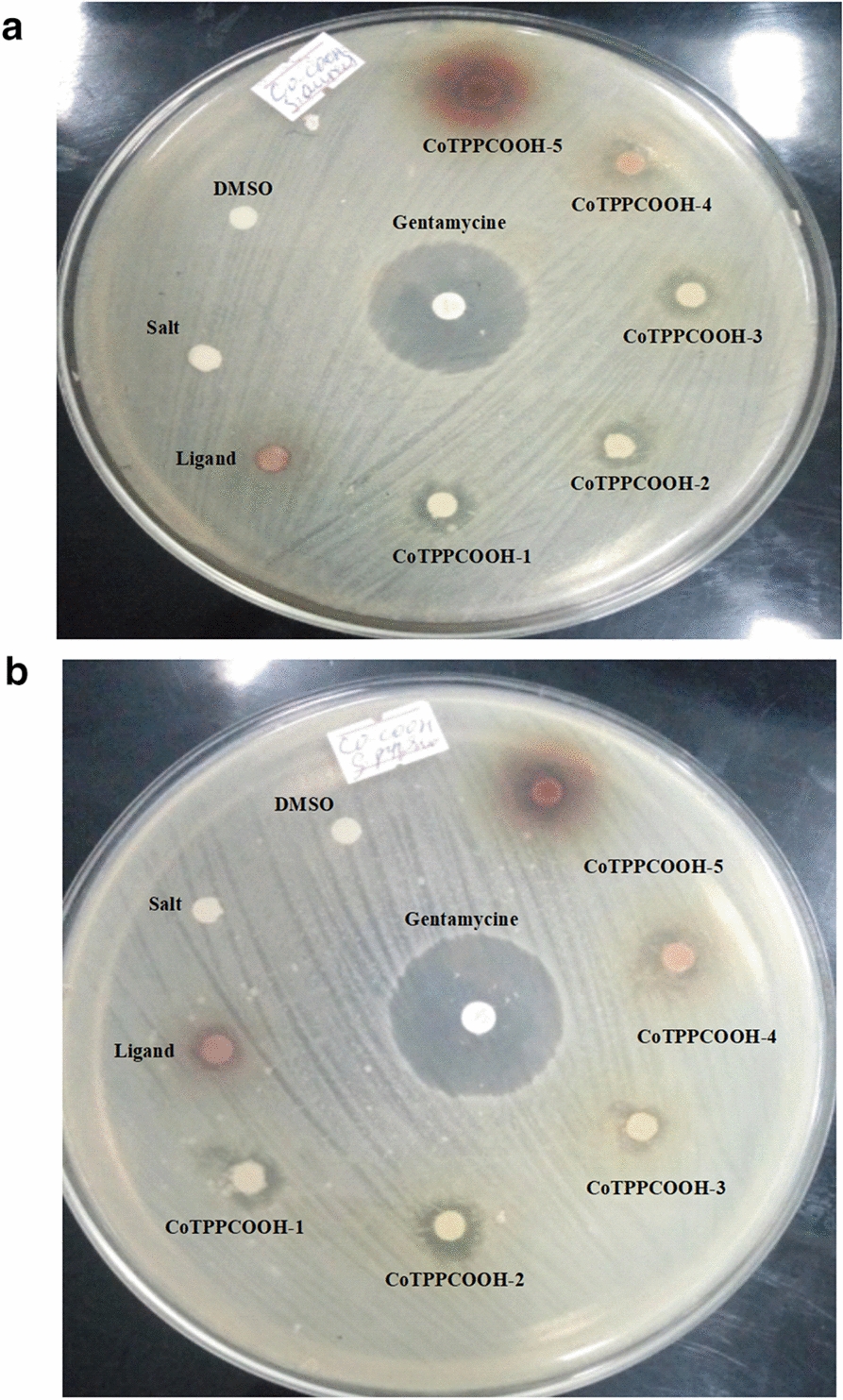
Inhibition of; **a** *Staphylococcus aureus* (*S. aureus*) and **b** *Streptococcus pyogenes* (*S. pyogenes*) by 5, 10, 15, 20-tetrakis (*p*-carboxyphenyl)porphyrinato cobalt (II)

**Fig. 4:**
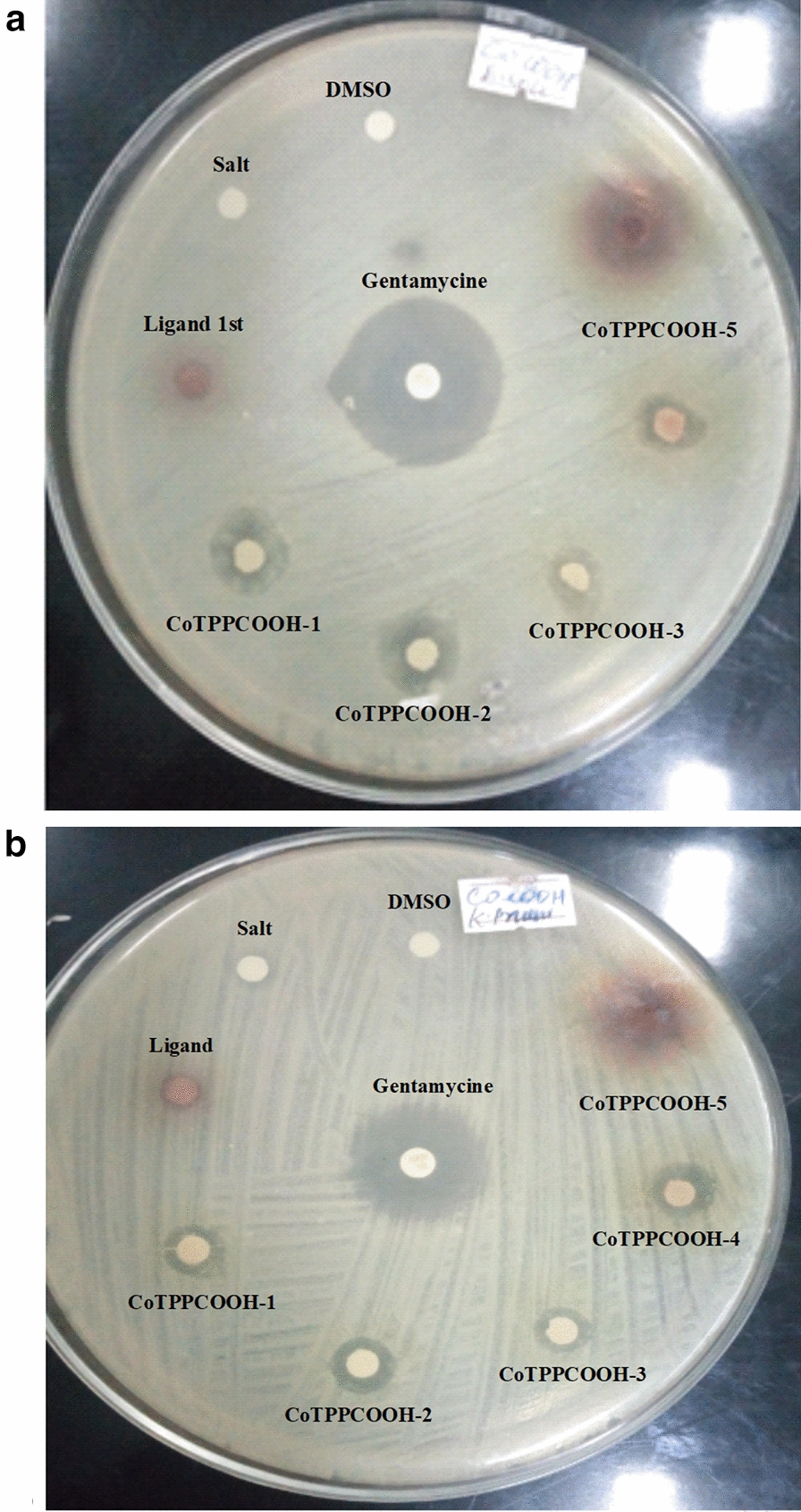
Inhibition of; **a** *Escherichia coli* (*E. coli*) and **b** *Klebsiella pneumoniae* (*K. pneumoniae*) by 5, 10, 15, 20-tetrakis (*p*-carboxyphenyl)porphyrinato cobalt (II)

For copper complexes of 5, 10, 15, 20-tetrakis (p-X phenyl)porphyrin, as the concentration of the complexes increase, the antimicrobial activity also increase as shown in Table 3 (Figs. 3, 4).

**Table 3:** Antibacterial activity (mean IZ diameter (mm) ± SD) of copperporphyrins, at different concentrations

Copper complexes at different concentration		Antibacterial activity (mean IZ diameter (mm) ± SD)			
		*S. aureus*	*S. pyogenes*	*E. coli*	*K. pneumonia*
500 mg/L					
CuTPPCOOMe		16 ± 0.2	15 ± 0.5	13 ± 0.6	13.5 ± 075
CuTPPNH_2_		13 ± 0.4	13.5 ± 0.3	12 ± 0.45	12.5 ± 0.6
250 mg/L					
CuTPPCOOMe		12 ± 0.45	12.5 ± 0.3	12 ± 0.02	11.5 ± 0.3
CuTPPNH_2_		11 ± 0.2	11 ± 0.6	10.5 ± 0.3	10 ± 0.2
125 mg/L					
CuTPPCOOMe		11.5 ± 0.5	11 ± 0.45	9.5 ± 0.02	10 ± 0.5
CuTPPNH_2_		10 ± 0.75	11.5 ± 0.4	9 ± 0.3	9.5 ± 0.4
62.5 mg/L					
CuTPPCOOMe		10.5 ± 0.2	10.25 ± 0.5	9 ± 0.03	9.5 ± 0.2
CuTPPNH_2_		9 ± 0.5	8.5 ± 0.5	9 ± 0.2	8.5 ± 0.45
31.25 mg/L					
CuTPPCOOMe		9 ± 0.4	8.75 ± 0.3	8.5 ± 0.04	8 ± 0.2
CuTPPNH_2_		8 ± 0.2	8 ± 0.1	7.5 ± 0.5	8 ± 0.75

Though antimicrobial activity of porphyrin derivatives of natural origin with COOH groups at *β*-pyrrolic positions have been reported so far [50–57], metalloporpyhrins with p-COOH at meso position of phenyl ring is not reported. Moreover, consistent with the report by Ke and coworkers, the electron withdrawing substituents enhance antibacterial activity attributing to increasing lipophilicity and polarity of the complex [15, 28]. Generally, the metal complexes containing electron withdrawal groups (with COOH and –COOMe showed better activities than the metal complex containing electron donating groups namely –NH_2_ and –OMe.

## Conclusion

In general, antibacterial activity of metalloprphyrins with different peripheral substituents is reported. The study indicated that all the complexes under study have promising antibacterial activity toward two Gram-positive (*Staphylococcus aureus *(*S. aureus*) and *Streptococcus pyogenes *(*S. pyogenes*) and two Gram-negative [*Escherichia coli *(*E. coli*)* and Klebsiella pneumoniae *(*K. pneumoniae*)] bacterial species. It is also found that bacterial growth inhibition by metallopophyrins is higher than the corresponding metal salt or DMSO. Increasing the concentration of the complexes slightly increases the inhibition activity. Among the complexes under study, the highest antibacterial activity is observed for CoTPPCOOH*,* which could be attributed to the high binding ability of COOH group to cellular components, membranes, proteins, and DNA as well as the lipohilicity of the complex. Moreover, consistent with literature report, the study revealed that metalloporphyrins with electron withdrawing group at para-positions have better antibacterial activity than metalloporphyrins which possess electron donating group at para position. The result finally concludes that metalloporphyrin derivatives are promising candidates for antibacterial activity.

## Abbreviations

DNA: Deoxyribonucleic acid
TPP: Tetra phenyl porphyrin
M (II): Metal with oxidation state of 2
Me: Methyl
OMe: Methoxy
COOMe: Methoxycarbonyl
ROS: Reactive oxygen species
°C: Degree centigrade
h: Hour
DMSO: Dimethylsulphoxide
IZ: Inhibition zone
SD: Standard Deviation
*S. aureus*: *Staphylococcus aureus*
*S. pyogenes*: *Streptococcus pyogenes*
*E. coli*: *Escherichia coli*
*K. pneumoniae*: *Klebsiella pneumonia*
g: Gram
mg: Milligram
L: Liter
mL: Milliliter
µg: Microgram
mm: Millimeter

## Availability of data and material

All data generated or analyzed during this study are included in this manuscript and its supplementary information files.
